# Azithromycin for Indigenous children with bronchiectasis: study protocol for a multi-centre randomized controlled trial

**DOI:** 10.1186/1471-2431-12-122

**Published:** 2012-08-14

**Authors:** Patricia C Valery, Peter S Morris, Keith Grimwood, Paul J Torzillo, Catherine A Byrnes, I Brent Masters, Paul A Bauert, Gabrielle B McCallum, Charmaine Mobberly, Anne B Chang

**Affiliations:** 1 Menzies School of Health Research, Charles Darwin University, Darwin, NT, Australia; 2 Department of Paediatrics, Royal Darwin Hospital, Darwin, Darwin, NT, Australia; 3 Queensland Children’s Medical Research Institute, The University of Queensland, Brisbane, QLD, Australia; 4 Queensland Paediatric Infectious Diseases Laboratory, Royal Children’s Hospital, Brisbane, QLD, Australia; 5 Royal Prince Alfred Hospital, and University of Sydney, Sydney, Australia; 6 Department of Paediatrics, University of Auckland, Auckland, New Zealand; 7 Paediatric Respiratory Medicine, Starship Children’s Health, Auckland, New Zealand; 8 Queensland Children’s Respiratory Centre, Royal Children’s Hospital, Brisbane, QLD, Australia

**Keywords:** Azithromycin, Bronchiectasis, Child, Chronic suppurative lung disease, Indigenous health, Placebo, Pulmonary exacerbation, Randomised controlled trial, Antibiotic resistance

## Abstract

**Background:**

The prevalence of chronic suppurative lung disease (CSLD) and bronchiectasis unrelated to cystic fibrosis (CF) among Indigenous children in Australia, New Zealand and Alaska is very high. Antibiotics are a major component of treatment and are used both on a short or long-term basis. One aim of long-term or maintenance antibiotics is to reduce the frequency of acute pulmonary exacerbations and symptoms. However, there are few studies investigating the efficacy of long-term antibiotic use for CSLD and non-CF bronchiectasis among children. This study tests the hypothesis that azithromycin administered once a week as maintenance antibiotic treatment will reduce the rate of pulmonary exacerbations in Indigenous children with bronchiectasis.

**Methods/design:**

We are conducting a multicentre, randomised, double-blind, placebo controlled clinical trial in Australia and New Zealand. Inclusion criteria are: Aboriginal, Torres Strait Islander, Maori or Pacific Island children aged 1 to 8 years, diagnosed with bronchiectasis (or probable bronchiectasis) with no underlying disease identified (such as CF or primary immunodeficiency), and having had at least one episode of pulmonary exacerbation in the last 12 months. After informed consent, children are randomised to receive either azithromycin (30 mg/kg once a week) or placebo (once a week) for 12–24 months from study entry. Primary outcomes are the rate of pulmonary exacerbations and time to pulmonary exacerbation determined by review of patient medical records. Secondary outcomes include length and severity of pulmonary exacerbation episodes, changes in growth, school loss, respiratory symptoms, forced expiratory volume in 1-second (FEV_1_; for children ≥6 years), and sputum characteristics. Safety endpoints include serious adverse events. Antibiotic resistance in respiratory bacterial pathogens colonising the nasopharynx is monitored. Data derived from medical records and clinical assessments every 3 to 4 months for up to 24 months from study entry are recorded on standardised forms.

**Discussion:**

Should this trial demonstrate that azithromycin is efficacious in reducing the number of pulmonary exacerbations, it will provide a much-needed rationale for the use of long-term antibiotics in the medical management of bronchiectasis in Indigenous children.

**Trial registration:**

Australian New Zealand Clinical Trials Registry: ACTRN12610000383066

## Background

Internationally, the burden of ill health from acute and chronic respiratory disease remains high in Indigenous populations [1-5]. While childhood chronic suppurative lung disease (CSLD), including bronchiectasis unrelated to underlying causes such as cystic fibrosis (CF) are much less common than a century ago, evidence is emerging of increasing prevalence in recent decades [6,7]. These chronic pulmonary disorders remain important in low and middle-income countries [8] and within disadvantaged population groups in high-income nations [9]. In these groups, bronchiectasis is associated with very high rates of childhood pneumonia and other acute respiratory infections (e.g. bronchiolitis) [10,11]. The prevalence of non-CF bronchiectasis among Indigenous children from remote communities in Australia is 1470/100,000 children [3]. Equivalent rates for Maori and Pacific Island children in New Zealand and for Alaska Native children from the Yukon Kuskokwim Delta region are 63, 154 and 1400–2000 per 100,000 children respectively [4,5]. In contrast, the estimated prevalence rates of bronchiectasis amongst New Zealand children under 15 years of age is 33 per 100,000 [5].

Bronchiectasis is a disease characterized by abnormal irreversible bronchial dilatation, which is associated with chronic bacterial infection and inflammation. ‘Symptoms’ include persistent ‘moist’ cough, mucopurulent sputum, haemoptysis, and breathlessness [2,12]. The current ‘gold standard’ for diagnosis is confirmation by a high-resolution computed tomography (HRCT) scan of the chest [13]. This investigation however, is not readily available in all settings. Bronchiectasis is considered within the spectrum of CSLD, which includes children with these same symptoms, but who lack HRCT scan evidence of bronchiectasis (either because of limited opportunity of testing or inability to meet the current HRCT scan adult criteria) [14]. Nonetheless, without treatment children with CSLD may continue to have persistent respiratory symptoms and progress to meet the radiological criteria for bronchiectasis [14]. Treatment aims at resolving acute infection and/or controlling established infection and inflammation, thus improving symptoms, reducing frequency of acute pulmonary exacerbations, preserving respiratory tissue and lung function, optimising growth, and improving quality of life (QoL) [14,15]. As bacteria such as *Haemophilus influenzae, Streptococcus pneumoniae* and *Moraxella catarrhalis* are believed to play a central role in the pathogenesis of CSLD and bronchiectasis [16] antibiotics are used to reduce the bacterial load and the associated lower airway inflammation [14]. Pulmonary exacerbations often require hospital-based treatment with intravenous antibiotics and intensive physiotherapy. Furthermore, severe pulmonary exacerbations are an independent risk factor for long-term lung function decline in children with bronchiectasis [17]. Long-term use of antibiotics may provide a benefit by reducing exacerbations, but are not recommended currently as part of routine treatment [14]. A recent Cochrane Review of 10 randomised, controlled trials (RCTs) involving 959 children and adults with CF reported a modest improvement in pulmonary function measured as forced expiratory volume in 1-second (FEV_1_) after 6 months of azithromycin, reduced pulmonary exacerbation rates and improved weight gains [18]. Azithromycin was well tolerated and while it was associated with decreased *Staphylococcus aureus* isolation rates in respiratory cultures, there was also a significant increase in macrolide resistance. In contrast, so far there are no published RCTs investigating the efficacy of long-term antibiotic use in children with non-CF bronchiectasis.

Following the methodological rigour outlined in the CONSORT statement, [19] we describe the methods of our Bronchiectasis Interventional Study (BIS), a multicentre, double-blind, RCT examining the efficacy and safety of maintenance azithromycin treatment (30 mg/kg once a week) versus placebo for 12–24 months in Indigenous children with non-CF bronchiectasis.

### Study aims

Our primary question is: among Indigenous children with bronchiectasis, can long-term (12–24 months) azithromycin treatment reduce the frequency of pulmonary exacerbations compared to placebo? Our secondary questions are: (i) does long-term azithromycin treatment reduce the length of hospitalised pulmonary exacerbations and severity of pulmonary exacerbations, improve growth, decrease school absenteeism, and improve respiratory symptoms, pulmonary function as measured by FEV_1_ (≥6 years), and sputum characteristics? (ii) is azithromycin associated with any serious adverse events (SAEs) or with increased antibiotic resistance in respiratory bacterial pathogens in the nasopharynx?

Our study tests the primary hypothesis that long-term (12–24 months) antibiotic treatment with azithromycin reduces the rate of pulmonary exacerbations in Indigenous children with non-CF bronchiectasis.

## Methods

### Participants and settings

This RCT is being conducted in Australia and New Zealand. Australian Aboriginal and Torres Strait Islander children from the Anangu Pitjantjatjara lands (Central Australia), the northern regions of the Northern Territory (NT) and the Torres Strait in Australia, and Maori and Pacific Island children from the greater Auckland region of New Zealand with bronchiectasis, are enrolled.

### Eligibility criteria

Inclusion criteria: (i) Self or caregiver ascribed Aboriginal, Torres Strait Islander, Maori or Pacific Island children, aged between 1 and 8 years; (ii) living currently within the study catchment community; (iii) with either a confirmed HRCT scan diagnosis of bronchiectasis or a clinical diagnosis of bronchiectasis (probable bronchiectasis, but no HRCT scan available) after clinical review and other appropriate investigations have been completed (including full blood count, immune function tests and sweat test, chest x-ray, and when indicated contrast-videoflouroscopy) [14,20] without a specific underlying cause identified e.g. CF, primary immunodeficiency, primary ciliary dyskinesia or primary aspiration, and (iv) having had at least one episode of pulmonary exacerbation in the last 12 months.

Exclusion criteria: (i) children receiving chemotherapy, immunosuppressive treatment or long-term antibiotic use, (ii) those with an identified underlying cause of their CSLD or bronchiectasis (e.g. CF, primary immunodeficiency, ciliary dyskinesia or primary aspiration), (iii) those with other disorders (e.g. renal or hepatic failure, diabetes, central nervous system or neuromuscular disorder, congenital cardiac abnormality), or (iv) a history of macrolide hypersensitivity.

If the local doctor (or paediatrician) decided that ‘long-term antibiotics or long-term placebo’ was not appropriate at the time (‘too well’ or ‘too sick’ to be randomised), the child was reassessed at a later stage and, if appropriate, was enrolled into the study at that time. Children already receiving long-term antibiotics may be randomised after they had discontinued antibiotics for at least 2 weeks.

### Recruitment

**Figure 1:**
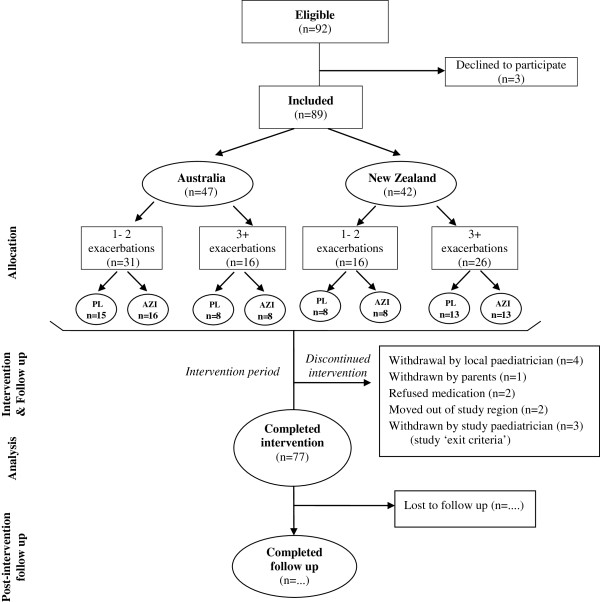
Participant flow diagram.

**Figure 2:**
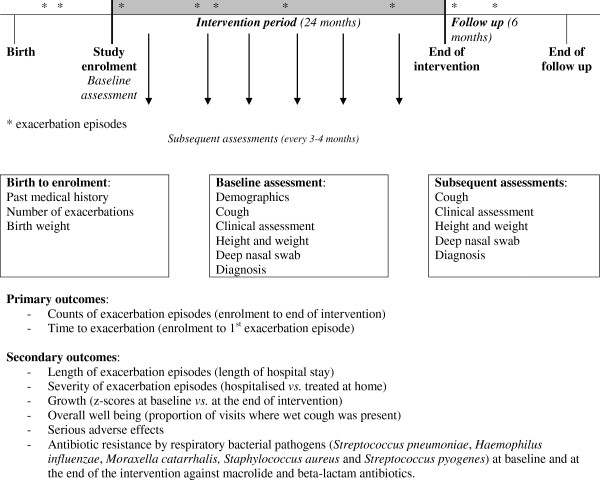
Outcome variables and time-points of assessment.

Caregivers of eligible children were approached and informed consent obtained between November 2008 and December 2010. The CONSORT flow diagram and the accompanying timelines, outcomes and procedures are displayed in Figures 1 and 2 respectively. At the end of the intervention period (12 to 24 months), children are followed for a further 6 months. Except for the number of children assessed for eligibility, the design is consistent with the CONSORT statement of 2010 [19] Unfortunately, due to the high prevalence of cough in Indigenous children in the communities included in the study, and the >2 year recruitment period, it was not feasible to collect information on all children assessed for eligibility. Follow-up assessments associated with the study are expected to be completed by late 2012.

### Follow-up schedule

Three full-time research nurses, based in Alice Springs and Darwin, NT, Australia, and Auckland, New Zealand, are responsible for the day-to-day management of study participants, and liaison with the co-ordinating centre based in Brisbane, Australia. Every 3–4 months, each study participant is asked to attend a study clinic. In Australia, clinics are held at local community clinics in 13 Indigenous communities (5 in the northern region of the NT, 6 in central Australia, 2 in the Torres Strait). In New Zealand, clinics are held at the Starship Children’s Health, Auckland City Hospital. Each subject receives at least 4 clinic reviews a year, including 2 by the study paediatrician and 2 by the research nurse.

### Subject efficacy withdrawal criteria

The following intervention ‘exit criteria’ (in case of ‘treatment failure’) are being used: the maximum numbers of pulmonary exacerbations allowed in any 12 month period are either 6 episodes treated by the local clinic (as an outpatient) or 4 hospital admissions. Children meeting the exit criteria are withdrawn from the intervention study, but continue to be followed until the end of the trial period. In addition, the local clinician or local investigator is able to withdraw a participant at any time. Children withdrawn from the study will not be replaced. Children exiting the study due to excess exacerbations are offered to go onto open label medication until the end of the study.

### Study medication

Azithromycin is purchased from Pfizer and rebottled by the Institute of Drug Technology (IDT, Melbourne). The placebo medication is also manufactured by IDT, Melbourne according to Australian standards and is similar in appearance, taste, smell and packaging to the active drug in order to maintain blinding during the study period. Prior to administering the trial medications, 9 mls of water is added to the bottle after piercing the aluminium foil making a total volume of 15 mls per bottle; 0.75 mls/kg (30 mg/kg/week for azithromycin) are given once weekly (maximum of 15mls). Each bottle is used only once and it and the remaining medication are then discarded.

### Randomisation, allocation and blinding

A computer generated permuted block design provided the randomisation sequences stratified by study site (Australia and New Zealand) and number of exacerbation episodes in the preceding 12 months (1–2 episodes vs. ≥3 episodes; Figure 1). A computer generated 3 digit number was used as the randomisation identification number. A treatment code (A to F) was allocated to each randomisation number. Only the independent statistician and IDT knew the codes. An independent person at the Queensland Institute of Medical Research (QIMR), Brisbane, Australia prepared individual envelopes labelled with the randomisation number and containing the corresponding treatment code inside. Allocation concealment was achieved by use of sequentially numbered sealed opaque envelopes. All treatment bottles are labelled identically for each child. Patients, their families, care providers and investigators collecting data are unaware of the treatment assigned to each child.

### Administration of the study medication and compliance

In Australia, Aboriginal and Torres Strait Islander children included in the study predominantly live in small remote communities of 200 to 1000 people. Study medication is administered under direct supervision at the community clinics. At numerous communities, local community workers are employed to locate the children weekly and dispense study medication. They contact the study nurse weekly to advise on the details about medication adherence (children receiving medication and, if any, children are absent from the community), and any issues around administration such as vomiting, “spitting up” the medication, abdominal pain or diarrhoea. These data are recorded in the ‘Participant Medication Log Book’.

In New Zealand, Maori and Pacific Island children included in the study mostly live in an urban environment in the greater Auckland region. Study medication is administered under direct supervision at the participant’s school, preschool or at their home. A whanau (support) worker is employed to assist with recruitment, informed consent, and to deliver and dispense study medication. The whanau worker records in the ‘Participant Medication Log Book’ the details about medication adherence and any issues around administration.

### Study endpoints

Primary outcomes: (i) rate of pulmonary exacerbations (treated as an outpatient or in hospital) and (ii) time to next pulmonary exacerbation. Each endpoint is determined by reviewing the patient and medical records.

Secondary outcomes: length of pulmonary exacerbation episodes; severity of pulmonary exacerbations (hospitalisation, oxygen requirement); change in growth (height, weight); respiratory symptoms (standardised questionnaire), sputum characteristics (a standardised and validated colour chart (Bronkotest colour chart) is used to grade sputum visually) [21], school absenteeism, FEV_1_ in those aged ≥ 6 years; and safety endpoints (serious adverse events (SAEs). Antibiotic resistance in respiratory bacterial pathogens detected in nasopharyngeal (deep nasal) swabs are also monitored.

Data are collected at 3 to 4 month intervals from medical records and clinical assessments (when possible) for up to 30 months from study entry (Figure 2).

### Outcome measurements and time point of assessments

The medical records of participating children (at the community clinic and the hospital) are monitored throughout the duration of the study. Information about primary study endpoints, other outcome measures, as well as follow-up visits (e.g. symptoms, clinical assessment, anthropometric measurements) are extracted from these records. Figure 2 lists the main outcome variables and the assessment time-points. Data are collected on standardised forms and entered into an Access database subject to internal range and logic checks to minimise errors. Data are then entered locally into a password-protected study database, on a secure website. An Australian-based data manager conducts regular data checks and queries to ensure data completeness and accuracy. Investigators and research nurses meet regularly to discuss data collection issues and study progress.

### Safety measurements

#### Serious adverse events

An independent Data Safety Monitoring Board (DSMB) was established to monitor data throughout the duration of the study and to determine whether it should be discontinued on scientific or ethical grounds. The DSMB monitors the clinical trial in accordance with the World Medical Association Declaration of Helsinki – Ethical Principles for Medical Research Involving Human Subjects, [22] as well as the Australian National Health and Medical Research Council National Statement on Ethical Conduct in Research Involving Humans (2007) and Values and Ethics: Guidelines of Ethical Conduct in Aboriginal and Torres Strait Islander Health Research [23].

SAEs include any untoward medical occurrence that results in death or is life threatening, results in significant disability/incapacity, requires inpatient hospitalisation or prolongation of existing hospitalisation. All SAEs are reported immediately by telephone to the Chief Investigator (CI) (study paediatrician in charge: PSM, Royal Darwin Hospital, Australia and CAB, Starship Children’s Health, New Zealand) and by fax to the Clinical Trial Coordinator (using a standardised SAE form). All SAEs are investigated by the CI in consultation with an Independent Safety Monitor (Dr Ngiare Brown, Australia and Dr Lesley Voss, New Zealand). Causality is classified and assigned as: Very likely/Certain, Probable, Possible, Unlikely, Unrelated, and Unclassifiable. SAEs with a causality classification of “very likely/certain”, “probable” or “possible” are reported to the Chair of the DSMB within 24 hours of their determination and the medication is withdrawn immediately, although the child will still remain in the trial. Other causality classifications are reported to the DSMB in summary format within 30 days, while all other adverse events are reported to CIs 3-monthly and are then submitted electronically to the DSMB Independent Safety Monitor.

### Bacteriology, including antibiotic resistance

At enrolment, and when possible at clinic reviews and hospital admissions, participants have a deep nasal swab collected as described previously [24]. Swabs of aural discharge are also taken if a discharging perforation of the eardrum is present. Swabs are placed into a tube containing 1 mL of skim-milk tryptone glucose glycerol broth (STGGB), [24] and are either transported frozen if taken during community visits or sent to the laboratory immediately on ice if collected while in hospital. All specimens are stored at -80°C until testing.

Culturing, identifying and serotyping common respiratory bacteria from nasal swabs are established techniques in our laboratory [24]. Batches of swabs are thawed and 10uL aliquots cultured overnight on selective media at 37°C in 5% CO_2_. *S. pneumoniae, H. influenzaeM. catarrhalisS. aureus* and *Streptococcus pyogenes* are identified using standard techniques [25]. *S. pneumoniae* isolates are serotyped using the Quellung method (antisera from Statens Serum Institute, Denmark).

Antibiotic susceptibility testing is undertaken initially on 4 colonies each of *S. pneumoniaeH. influenzaeS. pyogenes* and *S. aureus* using the calibrated disc sensitivity method [24]. When the inhibition zone indicates reduced antibiotic susceptibility to azithromycin, ampicillin and/or oxacillin, minimum inhibitory concentrations (MICs) are determined by Etest® strips (AB bioMérieux, Sweden) and resistance to azithromycin, erythromycin, penicillin and ampicillin as appropriate for the pathogen of interest is defined according to the European Committee on Antimicrobial Susceptibility Testing (EUCAST) breakpoints (http://www.eucast.org). A nitrocephin-based test detects beta-lactamase activity in *H. influenzae* and *M. catarrhalis* isolates.

### Sample size calculation

The study sample size and power calculations were based on our Central Australia data [11] where Indigenous children diagnosed with bronchiectasis had on average 1 pulmonary exacerbation every 6 months (standard deviation = 5.4) that required hospital admission. Thus, the ‘placebo’ group is expected to have 4 episodes involving hospital management during the 24-months of the trial intervention. Assuming the intervention reduces the number of pulmonary exacerbations leading to hospitalisation by 50% (from 4 to 2 episodes) and that the placebo group have a 15% reduction in hospitalised pulmonary exacerbations (from 4 to 3.4 episodes), then with 51 subjects in each group there is 95% power to detect an average difference of −1.4 respiratory hospital admissions per subject over a 2 year period between the intervention and placebo groups at the 5% level of significance. Importantly, these estimates used hospitalised exacerbation rates as a conservative estimate of total exacerbation rates (hospital management and those treated at the local clinic as outpatients). The sample size required to estimate the difference between the rate parameters of 2 Poisson distributions over 24 months with 90% power was 34 observations from each sample (68 child-years at risk in each group)**.**

### Data analysis

Data will be analysed using the statistical software package SPSS (IBM SPSS Statistics Version 20) and presented in accordance with the CONSORT statement [19]. All tests will be two-tailed, and statistical significance will be set at p <0.05. Study end points experienced for each treatment group will be formally analyse [26] on an "intention to treat" basis. Important potential confounders will be included in a secondary multivariate analysis. Basic descriptive summaries will be compiled for each treatment group. For continuous normally-distributed variables we will report means and compare groups using a *t*-test for independent samples. For non-normally-distributed data we will report medians and use non-parametric tests. If rates of exacerbation are consistent with a Poisson distribution, they will be assessed using Poisson regression. Categorical data will be analysed using the Chi-squared tests and Fisher’s exact test (2-tailed), as appropriate. Time to first exacerbation will be assessed using Cox proportional hazards regression and graphically displayed using Kaplan-Meier estimates. In addition, Epi Info (version 3.5.3) will be utilised to calculate growth z-scores. Children will be classified as stunted or underweight if their enrolment ‘height-for-age’ or ‘weight-for-age’ z-scores are less than −1.96, respectively, which indicate that the values are more than 2 standard deviations (SDs) below the corresponding population means.

### Ethical approval

Ethical approval has been obtained from the respective Human Ethics Committees of all participating institutions [Queensland Institute of Medical Research, Auckland District Health Board, the Northern Territories Department of Health and Community Services and Menzies School of Health Research, Central Australia Human Ethics Committee, and the Children's Health Queensland Hospital and Health Service].

## Discussion

Antibiotics are one of the main therapies used to treat bronchiectasis, both during acute pulmonary exacerbations, and in some patients as long-term maintenance treatment during the chronic stable phase. Based on Cole’s ‘vicious cycle’ model of the pathophysiology of bronchiectasis, [27] antibiotic treatments are recommended to reduce the bacterial load, which then decrease lower airway inflammation. Brief antibiotic interventions significantly improve airway [28,29] and systemic [28,29] inflammatory profiles, as well as improving QoL measures [29,30]. For the chronic stable phase, cohort studies [31,32] support the use of prolonged antibiotics for reducing exacerbations and sputum purulence, but there are only very limited data from RCTs to justify this approach [33].

The rationale for choosing azithromycin includes its recognised antimicrobial effects, its putative *in-vivo* immuno-modulatory and anti-inflammatory actions, and its unique pharmacological properties, which suggest once weekly oral dosing (30–40 hours half-life in children) may be effective [34-37]. In non-CF bronchiectasis, a cohort study [31] and one short term RCT (6 months) described improvement in lung function and reduction in pulmonary exacerbations when azithromycin was given to adults with bronchiectasis. In both studies, [31,38] the exacerbation frequency rate while taking regular azithromycin was significantly reduced (count ratio 0.5-0.7). However, to date there are no RCTs beyond 6 months and there are no studies of azithromycin in children with non-CF bronchiectasis.

The long half-life of azithromycin, allowing once weekly dosing is advantageous for adherence and even directly supervised administration, which is necessary for the feasibility and sustainability of long-term programmes involving maintenance antibiotic treatment in some settings. Any positive aspects of treatment must however be weighed against the possibility of increased antibiotic resistance amongst bacterial pathogens in the respiratory tract, particularly *H. influenzae, S. pneumoniaeS. pyogenes* and *S. aureus*[39]. The underlying mechanisms of macrolide resistance can confer either low or high-level resistance to individual members of this antibiotic class. Azithromycin is also associated with increased risk of resistance to other antibiotic classes, especially the beta-lactams, in respiratory bacterial flora [40]. While resistance is problematic and treatment failure has been reported, [41-43] macrolides still play an important role in managing many infectious diseases, including other respiratory infections, trachoma and sexually transmitted infections [44,45]. The current study is carefully monitoring antibiotic resistance in potential respiratory bacterial pathogens colonising the nasopharynx.

The reason for choosing pulmonary exacerbations as our primary outcome is two-fold. Firstly, in chronic respiratory disease (e.g. asthma and chronic obstructive pulmonary disease), pulmonary exacerbations are an important end point in clinical studies and most treatment strategies aim to reduce their frequency. Secondly, in a longitudinal cohort study in children with non-CF bronchiectasis, the only significant predictor of FEV_1_ decline (over 3-yrs) was frequency of hospitalised exacerbations [17]. With each exacerbation, the FEV_1_ % predicted decreased significantly by 1.95% adjusted for time. In adults with bronchiectasis, the determinants of accelerated lung function decline are frequency of hospitalised exacerbations, increased systemic inflammatory markers and colonisation with *P. aeruginosa*[46]. Increased mortality risk is also associated with the degree of lung function impairment [47]. Thus interventions that can reduce pulmonary exacerbations are likely to be important for preventing future adult lung dysfunction [48] in addition to reducing the economic and social costs associated with each episode [49]. Furthermore, recurrent exacerbations are one of the strongest predictors of poor QoL in adults with bronchiectasis [50]. This is consistent with data for asthma describing pulmonary exacerbations in childhood and adult asthma associated with accelerated FEV_1_ decline in those not receiving preventative therapy [51]. Thirdly, in children at this very young age respiratory exacerbations can be readily recorded, unlike some other outcome measures.

Strengths of our study include its randomised trial design, the inclusion of local community workers, and monitoring of adherence. This study is being undertaken in two very different settings: in Australia the study is being conducted mostly in remote, rural Indigenous communities with limited healthcare provision, while in New Zealand the study is being performed in a tertiary hospital in a large urban setting. The study design should help to reduce the potential limitation of heterogeneity in study settings by stratifying by site during randomisation and also by using standardised inclusion/exclusion criteria and data collection procedures and forms. Regular meetings between research personnel to discuss data collection issues and study progress should also help to ensure very good standardisation of the data collection procedures across study sites. The use of local community workers to aid recruitment, administer study medicine and address adherence directly will also assist in the successful conduct of the study. In Australia and New Zealand, study medication is administered to participants under direct supervision by either health clinic or study personnel, and the details about adherence and any issues around administration are carefully recorded on standard forms. This ensures good documentation of adherence with study medication.

A potential limitation of our study is that it is underpowered for small differences between groups. Nevertheless, even though our final enrolment of participants (n = 89) was less than the calculated target (n = 102), it still provides 90% power to detect statistically significant differences between the intervention and placebo groups.

Internationally, Indigenous children continue to have very high rates of chronic respiratory diseases, including bronchiectasis. If efficacious, a treatment regime of maintenance azithromycin to reduce the frequency of pulmonary exacerbations in Indigenous children with CSLD, including non-CF bronchiectasis is attractive as it is simple to administer It could substantially improve the prognosis of Indigenous children with bronchiectasis and would be a substantial advance in the treatment of these infants and children.

## Competing interests

The authors declare that they have no competing interests.

## Authors’ contributions

ABC, PCV, PSM, KG, PJT, CAB, IBM and PAB contributed to the study design. PCV participated in coordination, statistical analysis plan and drafted the manuscript. GBM and CM participated in coordination and data acquisition. ABC, PSM, CAB, GBM and CM actively recruited participants. All authors contributed to the writing of the manuscript and approved its final version.

## Pre-publication history

The pre-publication history for this paper can be accessed here:

http://www.biomedcentral.com/1471-2431/12/122/prepub
